# Implementation of Group Interpersonal Psychotherapy in primary care

**DOI:** 10.11606/s1518-8787.2022056003731

**Published:** 2022-04-11

**Authors:** Maria Isabel Perez Mattos, Bruno Paz Mosqueiro, Scott Stuart, Giovanni Salum, Rosana de Lima Duzzo, Laura Wolf de Souza, Ariane Chini, Marcelo Pio de Almeida Fleck

**Affiliations:** I Universidade Federal do Rio Grande do Sul Hospital de Clínicas de Porto Alegre Departamento de Psiquiatria e Medicina Legal Porto Alegre RS Brasil Universidade Federal do Rio Grande do Sul. Hospital de Clínicas de Porto Alegre. Departamento de Psiquiatria e Medicina Legal. Porto Alegre, RS, Brasil; II University of Southern California Psychiatry Department Los Angeles California United States of America University of Southern California. Psychiatry Department. Los Angeles, California, United States of America; III Prefeitura Municipal de Porto Alegre Unidade de Atenção Primária à Saúde Porto Alegre RS Brasil Prefeitura Municipal de Porto Alegre. Unidade de Atenção Primária à Saúde. Porto Alegre, RS, Brasil

**Keywords:** Health Personnel, Psychology, Primary Health Care, Group Interpersonal Psychotherapy, Communication Barriers, Health Plan Implementation

## Abstract

**OBJECTIVE:**

To show the implementation process of IPT-G in primary care, including facilitating and obstructing factors, implementation strategies, and training and supervision of primary care professionals.

**METHODS:**

Quantitative (cross-sectional and longitudinal) analysis of pre and post-knowledge tests; qualitative analyses of the training courses; patient recruitment; conduction of IPT-G sessions; supervision of IPT-G therapists; application of a semi-structured questionnaire to assess, investigate, and develop strategies against the identified barriers.

**RESULTS:**

About 120 clinicians answered the pre-test; 84 completed the post-test. Pre- and post-test scores of IPT-G knowledge were significantly different. Twenty initially trained clinicians completed additional supervision in IPT-G. Qualitative analysis identified twelve barriers and six facilitators to IPT-G implementation in individual, organizational, and systemic contexts.

**CONCLUSIONS:**

Implementation of IPT-G in primary care is a complex process with several steps. In the first step, health professionals were successfully trained in IPT-G. However, subsequent steps were more complex. Therefore, careful planning of IPT-G implementation is essential to maximize the success of this innovation.

## INTRODUCTION

Most guidelines recommend psychotherapy for the treatment of mild and moderate depression and medication with psychotherapy for severe depression^1^. Several meta-analyses^2^conclude that psychological therapies, including those offered in primary healthcare, are effective against depression.

Interpersonal psychotherapy (IPT) is a first-line treatment in primary healthcare because it is brief, manualized, and cost-effective^1,7,8^. Moreover, it can be applied by health professionals who are not specialists in mental health^5,9,10^. The IPT was first adapted by Denise Wilfley and Roy MacKenzie for group therapy (IPT-G) to manage eating disorders^11^, and it could be even more cost-effective as a group intervention^11^.

Weissman^10^ emphasizes that, especially in countries with limited mental health resources, community health workers should receive IPT-G training. In Uganda, for example, the IPT-G conducted in primary care by community health agents not specialized in mental health was more effective than treatment as usual^14^.

Therefore, if primary care professionals are adequately trained in IPT-G, they can significantly affect the healthcare of depressed patients^15,16^. Evidence shows that primary healthcare professionals are interested in obtaining psychotherapy training^15^. Ramanuj et al.^8^ showed that patients showed fewer depressive symptoms after such professionals were trained in more complex interventions.

Despite evidence-based benefits of psychological treatments to low- and middle-income countries, few national health systems use psychotherapies as a care option^14^. In part, this is likely because professionals do not receive psychotherapy training^7^. In Brazil, for instance, medication is still the main treatment for depression in primary care^17^. However, evidence shows that most patients prefer psychotherapy to pharmacotherapy, especially in primary care settings and in cases of depression and anxiety^18,19^.

With training, brief psychotherapies can become effective treatments. The IPT identifies and treats psychosocial problems associated with depressive symptoms and could potentially benefit populations with high rates of psychosocial stressors, including those from middle- and low-income countries such as Brazil^19^.

The diffusion of innovations depends on scientific evidence and many other factors^20^. Implementation science^20,22,23^is a relatively new area of research in health that can be defined as “[...] the scientific study of methods that promote the systematic introduction of research findings and other evidence-based practices into routine practices and thus improve the quality and effectiveness of health services”^24^. Thus, in implementation science, the context in which an innovation is introduced should first be understood^25^ instead of simply controlled or tolerated as in efficacy or effectiveness trials.

After identifying the lack of psychosocial approaches to depression, the coordination of the mental health service of the public primary care network in Porto Alegre sought a partnership with the Department of Psychiatry and Behavioral Sciences of the Universidade Federal do Rio Grande do Sul (UFRGS). They aimed to develop alternatives to drug treatment using evidence-based psychotherapies, expecting to reach many patients.

This study aims to: a) Describe the training of primary care professionals for IPT-G performance; b) Assess if training can effectively increase IPT-G proficiency of health professionals, using a pre- and post-knowledge test analysis; c) Describe the difficulties of IPT-G implementation in public primary health care; and d) Present implementation strategies that can contribute to new studies and processes for innovation in health services.

## METHODS

This project was carried out with a partnership between the Municipal Health Department (SMS) of the municipality of Porto Alegre and the postgraduate course in Psychiatry and Behavioral Sciences at UFRGS. The Primary Health Care and Health Policies Department (DAPPS) approved the use of data for health professionals. All data were anonymously used with consent of the Health Department of Porto Alegre. No specific consent form was given to any participant. However, after participants completed the enrollment form, they were informed that the DAPPS authorized anonymous use of all implementation process data in scientific publications.

In 2019, a training course aimed at providing basic knowledge of Interpersonal Group Therapy (IPT-G) was proposed for the qualification of general and mental health professionals, especially those in primary care.

In total, 1,194 health professionals were invited to the course and 140 of them accepted the invitation.

This study used a mixed methodology, combining quantitative (cross-sectional and longitudinal design) and qualitative research methods to describe and analyze data.

Participants were health professionals (nurses, psychologists, psychiatrists, community health agents, health technicians and residents) working in primary care at the healthcare centers (UBS) of the city of Porto Alegre (Table 1).

**Table 1:** Sample characteristics (n = 120).

Age	
Mean (SD)	39.96 (10.71)
Minimum/maximum	20/64
Gender n (%)	
Female	107 (89%)
Male	13 (11%)
Professionals n (%)	
Nurses	36 (30%)
Psychologists and psychiatrists	19 (16%)
Other health professionals^a^	18 (15%)
Community agents	17 (14%)
Health technicians^b^	13 (11%)
Non-psychiatric physicians	11 (9%)
Residents	6 (5%)

^a^ Dentists, nutritionists, speech therapists, physiotherapists, occupational therapists, and social workers.

^b^ Nursing technicians and oral health assistants.

Health professionals were recruited by invitations sent via email and by advertisements about the training program posted in each primary care unit. Participation was optional and the training time was paid as working hours.

Those who chose to participate signed up and completed a form for demographic information collection. They also answered one qualitative open question about their motivation for training and the responses were organized in categories (Table 2).

**Table 2:** Comparison of the baseline knowledge test results and the gross and adjusted changes between participant characteristics.

Features	Pre-test mean (SD)	Mean change^b^ (DP)	Adjusted mean change^c^ (95%CI)
Sex			
Female	8.1 (2.5)	4.2 (3.9)	4.2 (3.4–4.9)
Male	8.5 (3.1)	0.3 (3.1)	0.7 (-3–4,4)
Statistical test / p-value	T[gl = 118] = -0.634/ p = 0.527		F(gl = 1.81) = 3.395/ p = 0.069
Age			
20–29	8.5 (2.5)	4.8 (3.6)	5.5 (3.3–7.6)
30–39	9.1 (2.4)	3.0 (3.8)	4.3 (3.0–5.5)
40–49	6.7 (2.5)^a^	4.6 (3.9)	3.3 (1.9–4.6)
≥ 50	7.6 (2.4)^a^	4.5 (4.2)	3.9 (2.5–5.4)
Statistical test / p-value	F(gl = 3.116) = 6.641/ p < 0.001		F(gl = 3.79) = 0.983/ p = 0.405
Professional category			
Psychologists and Psychiatrists	8.6 (2.4)	3.7 (4.9)	3.8 (1.8–5.7)
Non-psychiatric physicians	7.5 (3.5)	5.4 (2.9)	4,9 (2.4–7.4)
Nurses	8.4 (2.3)	3.9 (3.6)	4.2 (2.8–5.6)
Graduate health professionals	8.1 (2.6)	3.6 (4.7)	3.9 (2.1–5.6)
Health technicians	7.7 (2.9)	3.8 (4.5)	3.7 (1.7–5.7)
Community workers	7.6 (2.8)	4,9 (3.2)	4.3 (2.4–6.2)
Residents	8.5 (2.1)	2.5 (1.3)	2.7 (-0.6–6.0)
Statistical test/p-value	F(gl = 6.113) = 0.437/ p = 0.853		F(gl = 6.76) = 0.244/ p = 0.960

^a^ Means with statistically significant difference in relation to the 30–39 group by the Tukey test (α = 0.05).

^b^ Difference between pre- and post-test.

^c^ Results obtained by ANCOVA, adjusted by the baseline value of the knowledge test.

The IPT-G implementation project was divided into five phases: a) Training of registered professionals; b) Identification of professionals interested in applying the IPT-G in their UBS; c) Supervision of professionals qualified and interested in the implementation; d) Application of IPT-G to groups of primary care patients; and e) Completion of a semi-structured questionnaire by participating health professionals to assess the IPT-G implementation process.

The IPT-G trainer is a psychologist and a doctoral student in Psychiatry and Behavioral Sciences at UFRGS. The trainer was certified in IPT-G by the IPT Institute (USA). In 2019, a training course aimed at providing basic knowledge of Group Interpersonal Therapy (IPT-G) was proposed for the qualification of general and mental health professionals, especially those in primary care units.

The course was divided into 5 weekly meetings (3 hours each; 15 hours total), with the following agenda: a) Introduction to IPT-G; b) IPT-G problem areas (grief, transitional roles, and interpersonal disputes); c) Pre-group individual assessment; d) Interventions and management of the group e) Individual post-group interview; f) Implementation.

The teaching methodologies used were expository classes with audiovisual support, the practice of “role-play”, and illustrative fictional sessions in IPT-G.

Participants were qualified for basic training in IPT-G if they attended all classes and completed a pre- and post-knowledge test.

The knowledge test consisted of 25 multiple-choice questions translated from IPT Institute’s assessment test for IPT-G certification.

The supervision phase began after the training classes. All professionals who qualified for basic training were encouraged to start applying the IPT-G technique under supervision. Participation was optional but paid as working hours. Monthly supervision was offered to all potential group coordinators so they could learn both with their own groups and from the experiences of their peers. Besides in-person supervision, a remote group contact was also available by a messaging service.

Following the technique proposed by Stuart^13^, the IPT-G was planned to be used with patients with depressive symptoms in the primary care units where the selected professionals worked. The IPT-G treatment included 12 weekly sessions of 75 minutes each.

After IPT-G supervision ended, clinicians were invited to complete a semi-structured questionnaire created by the researchers. This questionnaire included seven open questions that required written responses regarding the main facilitating or obstructing factors for IPT-G implementation and strategies for implementation in the municipal health care service. Content analysis of the data was conducted with category-systematized findings.

### Statistical Analysis

Descriptive analyses of the demographic characteristics of participants were carried out using absolute and relative frequencies. Additional tests were performed to assess the participants’ performance in the qualification test. The Shapiro-Wilks test was used to verify the normal distribution of the number of correct answers, described by the mean and standard deviation. The paired sample t-test was used to compare changes in the performance of pre-training and post-training participants in a 25-question knowledge test. Baseline knowledge was compared using an independent t-test and analysis of variances (ANOVA) according to participant characteristics. The difference (delta) between pre- and post-training was compared for the different characteristics of the sample by the analysis of covariance (ANCOVA) with adjustment for baseline values. The level of significance for all analyzes was 0.05. SPSS software version 18.0 was used for all statistical analyses.

## RESULTS

Around 11.7% (140/1194) of the 1,194 health professionals invited enrolled in the IPT-G training course; 120 completed the pre-test, which was optional, and the other 20 declined. In total, 84 professionals participated in the five training sessions and completed the post-test, also optional.

Most participants were female (89%) and 11% were male. These participants were classified as nurses (30%), psychologists and psychiatrists (16%), and other health care professionals (54%). For result presentation, participants were distributed into seven groups (Table 1).

Participants increased their scores from 8.2 (SD = 2.8) in the pre-knowledge test to 12.2 (SD = 3.3) in the post-knowledge test. This difference was statistically significant (t [gl = 83] = - 9.421; p < 0.001), with a 4.02 difference between means (95%CI: 3.17–4.9) (Table 2).

Participants in the 30–39 age group performed better in the knowledge pre-test, which had significantly different results based on age. However, analysis of covariance showed that in the post-test the groups had no significant difference regarding age and professional categories (Figure).

**Figure:**
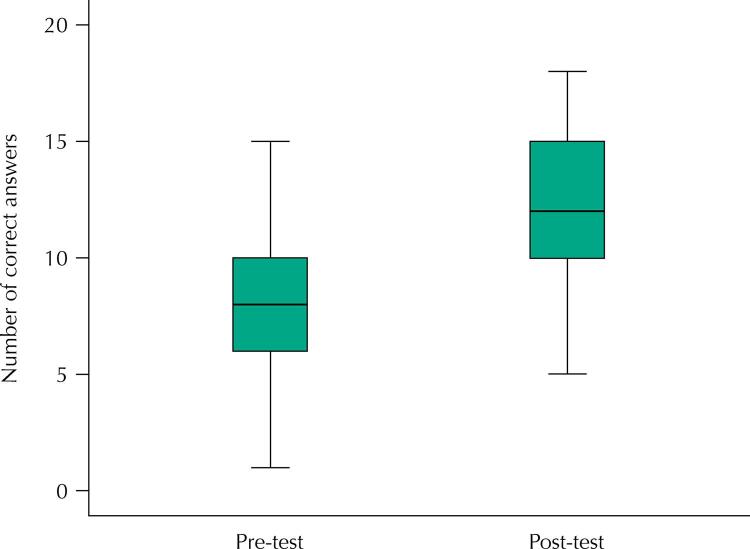
Number of correct answers in the pre- and post-training knowledge test.

Patients described motivations to participate in training, which were grouped into 10 empirical categories (Table 3). The main reason, mentioned by 68 participants (56.7%), was to increase knowledge about the technique and about Interpersonal Psychotherapy. Moreover, 35 participants (29.2%) wished to apply this group service in their units.

**Table 3:** List of main motivations for training.

Item	n (%)
To expand knowledge about interpersonal therapy	68 (57%)
Implement group care at respective unit	35 (29%)
Professional qualification	27 (23%)
To handle situations with patients with mental disorders	26 (22%)
To qualify for application in primary health care	18 (15%)
Matrixing	6 (5%)
Personal qualification	5 (4%)
To have more tools to welcome users	5 (4%)
To decrease demand for medical appointments	1 (1%)
Pharmacological management	1 (1%)

Each of three IPT groups started training under one coordinator. New groups were scheduled to start following the supervision period; however, at the second supervision meeting, participants brought up the potential dismissal of health professionals, which would affect most individuals enrolled in the IPT-G training. Their distress was discussed in supervision meetings, and this dismissal became a substantial obstacle to IPT-G implementation.

Thus, implementation was interrupted because of the potential dismissal of most professionals who attended the course after unexpected changes in the structure of the municipality’s health system at the time of supervision. A judicial decision discontinued the Municipal Institute of Family Health Strategy (IMESF), which employed many of the health professionals in Porto Alegre. As a result, 1,194 employees (doctors, community health and endemic disease control agents, nursing technicians, nurses, dentists, dental assistants, and oral health technicians) were dismissed so new professionals could be hired under another labor regime. Supervisors reported signs of distress of professionals who could be affected by this change, including interruptions and uncertainties in work. Moreover, other barriers to IPT-G implementation were identified. Over half (56.7%) of the participants indicated that they attended training to expand their knowledge about the IPT-G, whereas only 29.2% wished to apply IPT-G in primary healthcare.

One group session was carried out during monthly supervision. This group included three adolescents who showed depressive symptoms and self-mutilated. They were supervised by a psychologist who was not affected by the dismissal situation. Clinicians progressively quit supervision: 20 attended the first supervised session, but only 10 attended the last session.

Responses to the semi-structured questionnaire were organized into three main categories: facilitators, barriers, and suggestions for the implementation process. Sub-categories were identified in each of the main categories.

The following implementation facilitators were identified and placed in sub-categories: funding of the scientific partnership initiative with the university; engagement of the Municipal Mental Health Coordinator; provision of adequate space for the course; and availability of paid time for students and clinicians to attend training.

The identified barriers were grouped in three dimensions: professional, organizational, and systematic. Suggestions for strategic alternatives to overcome barriers were described by researchers and grouped in the same three dimensions (Table 4).

**Table 4:** Barriers and implementation strategies.

Barriers	Implementation strategies
**Professionals**	
Different contexts in the Primary Care Units	• Adjust the methodology to the context: expand application to different diagnostic groups;(e.g.: adolescents who self-mutilate, anxious patients).
Belief that open groups are easier	• Study the effectiveness of the open group; • Propose IPT-G experience (closed group) for assessment and results; • Create a culture of result assessment.
Perception of work overload	• Assess perceived or real overload; • Familiarize professionals with IPT-G practice by continuous supervision; • Include IPT-G in the activities agenda of professionals.
Inadequate physical space for care	• Assess available and alternative spaces for group care; • Adjust group size to the available space; • Include coordinators of each unit in this decision.
**Organization**	
Lack of support, integration, and co-participation of the broader network and local administrations	• Include managers in project discussion meetings; • Create a culture of result assessment.
Absence of surveys regarding previous needs of each unit	• Discuss and adapt IPT-G to the needs of each unit.
Short-term supervision	• Enable long-term supervision; • Train supervisors within the public network.
Occupational instability (dismissals caused by changes in the work regime)	• Forecast incentives and benefits for professionals involved in IPT-G; • Connect the team and the manager to the IPT-G project; • Manage the team at each unit to implement innovative projects.
Change of the municipality’s mental health coordinator	• Maintain long-term policies that remain even after the change of coordination; • Include and motivate the network, including teams and different levels of management.
**System**	
Immediate care culture	• Create a culture of result assessment and feedback to improve interventions.
Public policy instability	• Develop evidence-based public policies (e.g.: IPT-G); • Monitor implementation at long term seeking consolidation.
Suspension of the IPT-G project because of the covid-19 pandemic	• Resume the project after the pandemic is controlled; • Use virtual communication technology (IPT-G Remote); • Provide Internet access for most users; • Incorporate new remote technologies as a potential additional resource.

Data processing required 18 months between training disclosure until application of instruments.

## DISCUSSION

Although the participants’ low performance suggests that the test was difficult, the training course had a statistically significant effect on their levels of IPT-G knowledge. We did not use a cut-off score for approval. The test was originally developed to assess a training course for health professionals who attended level A of the IPT Institute (USA).

All professional categories equally improved their test performance, which shows that IPT-G training is effective regardless of previous training. This finding corroborates to studies that indicate the potential of professionals from different areas of health and of community health agents to carry out psychological interventions such as IPT-G, especially in low-income countries, which need more interventions and mental health professionals^5,9,10,12,14,26^.

Responses to the questionnaire about facilitators, barriers, and suggestions for IPT-G implementation indicated that participants were motivated and in fact learning the IPT-G technique. Furthermore, they emphasized how much users could benefit from a non-pharmacological technique for the treatment of depression.

The barriers to IPT-G implementation seem unrelated to the course itself or to its modality (IPT-G). We emphasize that the only completed IPT Group was supervised by a professional who was not fired after the political decision to extinguish IMESF. Araújo et al.^26^ identified that training alone seems unable to produce lasting effects on the participant’s behavior since it depends on the post-training context. Psychosocial support for immediate use was confirmed as the strongest predictor of training impact. It involves the support offered by managers, co-workers, and the organization so that the professional can participate in training and then apply the skills learned^26^.

Therefore, planned implementation strategies developed post-training can make the course effective^26^.

Mutamba et al.^14^ found noteworthy effectiveness data on IPT-G implementation in Uganda: 90% of users completed the IPT-G program and the prevalence of mental health problems decreased. In their study, participants in training were not mental health specialists, but members of the community. The results were attributed to the implementation process. Strategies included logistics and technical advice with government working groups who managed public policies.

In our study, although most trained professionals were also not specialized in mental health, they acquired knowledge with the training process. However, the articulation and integration between work groups and primary healthcare service stakeholders was insufficient to implement public policies for innovations like IPT-G.

Although professionals felt initially motivated for training, IPT-G implementation was affected by several factors. The identified barriers can be attributed to factors of three levels: a) Professional (individual), b) Organizational (management, health unit to which the professional is connected), and c) Systemic (public health policies). Strategies were created to reduce these barriers and increase the effectiveness of IPT-G implementation (Table 4).

According to implementation science, innovations are consolidated by a project-execution-analysis-action cycle, which includes constant monitoring, evaluation, and feedback, besides financing, public policies, and management support. Thus, the organizational culture can change and innovate^21^.

However, researchers did not sufficiently predict implementation barriers, including the legal decision which dismissed most trained professionals. Moreover, we observed scheduling problems, inadequate physical space, poor integration of the IPT-G initiative with other initiatives of the primary healthcare services, and a lack of coordination between each unit and the Municipal Health Department. Table 4 lists barriers and implementation suggestions such as a greater integration of the different levels in management, the possibility of adapting the IPT-G to the context of each unit, and the need for structural improvements.

Because of the covid-19 pandemic, we could not immediately resume an implementation strategy for the identified barriers.

The IPT-G training project presented in this study was an innovative experience to qualify health professionals for the application of an effective psychotherapeutic technique for the treatment of depression in primary care^1,6,7,13^. However, implementation was affected by various barriers. Our study could be the basis for a new initiative for the implementation of IPT-G or other strategies in public health.

### STUDY LIMITATIONS

This study involved a limited number of professionals in a single city. Thus, its findings cannot be applied to other contexts. After the initial course, few professionals were involved in the recruitment and execution of groups and few accepted to participate in the semi-structured interviews to identify barriers and facilitators. Although this limited us from obtaining information, we found that involving several professionals in innovative projects is difficult. Furthermore, our study was also limited by unique events of the health system restructuring and the covid-19 crisis. Despite its limitations, this study is original and relevant for understanding the training process and possible barriers in the implementation of new mental health treatment strategies in primary care.

## CONCLUSIONS

This study describes an attempt of IPT-G implementation process in public primary healthcare.

The IPT-G implementation in primary care is a complex process with several steps. The first step relatively increased the health professionals’ knowledge of IPT-G. However, subsequent steps were more complex. Some of the barriers and impediments were unexpected, such as the dismissal of participating professionals.

To effectively implement the IPT-G in primary healthcare, we must offer training to healthcare professionals and plan several steps and strategies based on implementation science. Therefore, careful planning can anticipate difficulties and result in a well-articulated project, which will lead stakeholders and teams to help achieve positive results.

Stable public policies that are cost-effective and value evidence-based effective implementation measures are essential to identify and remove barriers by implementation science.
